# Agreement and internal quality assurance of the Neubauer hemocytometer and Makler chamber for human sperm concentration determination

**DOI:** 10.5935/1518-0557.20240023

**Published:** 2024

**Authors:** Ane Francyne Costa, Fabiana Botelho de Miranda Onofre, Alexandre Sherlley Casimiro Onofre

**Affiliations:** 1 Department of Clinical Analysis, Federal University of Santa Catarina, Florianópolis, Brazil

**Keywords:** sperm concentration, Neubauer hemocytometer, Makler chamber, infertility investigation, latex beads

## Abstract

**Objective:**

The Neubauer hemocytometer, as well as the Makler chamber, are devices commonly used in andrology laboratories. The present study aimed to verify if both methods yield comparable results, and whether they can be used interchangeably to determine sperm concentration.

**Methods:**

Sperm and latex beads concentration measurements were performed with the Neubauer hemocytometer and the Makler chamber. Fixed and proportional biases were estimated, and the method agreement was determined by assessing sperm concentration results with the Bland and Altman plot. The Coefficient of Variation (CV) and relative bias were calculated as an index of precision and accuracy, respectively, by measuring latex beads target concentrations in both chambers.

**Results:**

The Makler chamber systematically overestimated the Neubauer hemocytometer concentration measurements by a mean of -7.99%, with limits of agreement (LOA) between -41% to 25.61% (*p*<0.001). The fixed bias was found for concentration values inferior to 40 x 10^6^/ml range (*p*<0.001), but not higher concentration results (*p*>0.05). Measurements with the Neubauer hemocytometer showed the greatest consistency in the study with the CV ranging from 3.01% to 6.67%; while the CV with the Makler chamber ranged from 8.46% to 25.64%. The relative bias for the Neubauer hemocytometer determinations varied from 0.12% to 8.40%, while for the Makler chamber varied from 7.6% to an overestimation of 38.0%.

**Conclusions:**

Measurements made with the Makler chamber demonstrated more variability and a higher degree of overestimation. The Makler chamber is a poor substitute to the Neubauer hemocytometer for evaluation of oligozoospermic samples, although both chambers render similar results for highly concentrated samples.

## INTRODUCTION

Infertility is a global reproductive problem faced by 8 to 12% of couples (Vander Borght & Wyns, 2018); and about 30% of infertility cases are due to male factors alone (Agarwal *et al*., 2015). Precise semen analysis is crucial as there is growing concern that the sperm concentration of men in the general population has been declining over the last years (Carlsen *et al*., 1992; Levine *et al*., 2017; 2023; Swan *et al*., 2000). A variety of commercially available methods exist to assess sperm concentration, but lack of quality control and standardization may lead to discrepancies in results and, consequently, misdiagnosis and misguided selection of treatment strategy in the clinical setting (Dardmeh *et al*., 2021; Peng *et al*., 2015).

The World Health Organization (WHO) recommends the use of the Neubauer hemocytometer (NH) for sperm concentration assessment (WHO, 2021). Other counting devices must first be validated. The Makler chamber (MC) was first introduced in 1978 as an alternative to facilitate sperm count because assessment could be done rapidly and directly from an undiluted sample (Makler, 1978). In an external quality assurance program in Spain (Alvarez *et al*., 2005), 100% of the laboratories reported using the Makler chamber in 1989, and 67.5% remained using the chamber by the end of their study in 2002. In China (Wang *et al*., 2022), they used the Makler chamber steadily from 2009 to 2020 (33.0% to 37.0%). In a retrospective study in Brazil (Sertão & Machado, 2019), a total of 273 laboratories were enrolled in external quality assurance programs for semen analysis from 2016 to 2017. The highest percentage of error was found for sperm concentration results, ranging from 12.6% to 20.7%. However, it was not possible to discriminate which method was used in the reports. Therefore, there is a need to implement stricter quality control programs for concentration analyses.

Sperm counts with the Makler chamber have been shown to be generally higher than those obtained by the Neubauer hemocytometer in a few studies (Bailey *et al*., 2007; Dardmeh *et al*., 2021; Ginsburg & Armant, 1990; Peng *et al*., 2015; Seaman *et al*., 1996), although sperm counts demonstrated good agreement between both chambers in other studies (Cardona-Maya *et al*., 2008; Marchlewska *et al*., 2010). Conversely, there has been poor (Christensen *et al*., 2005; Zuvela & Matson, 2020) and acceptable (Imade *et al*., 1993) precision for the Makler chamber. Well-established internal quality control is imperative to detecting errors, thus, improving the quality of laboratory assessments and external quality results (Kinns *et al*., 2013). Therefore, the purpose of this study was to comparatively assess the agreement between the Neubauer hemocytometer and the Makler chamber human sperm concentration measurements as well as to evaluate the precision and accuracy of these two methods by measuring latex beads target concentrations for internal quality assurance.

## MATERIALS AND METHODS

### Study Population and sample collection

Adult male volunteers were recruited between Abril and September 2022 as part of an investigation of semen quality among students, professors, and maintenance staff of the Federal University of Santa Catarina (Florianópolis, Santa Catarina, Brazil). This study was approved by the Research Ethics Committee of the Federal University of Santa Catarina and given certificate number 19806819.1.0000.0121. Written informed consent was obtained from each participant.

In total, 221 semen samples were obtained consecutively from volunteers between the ages of 18 to 63 years at the University Hospital of the Federal University of Santa Catarina. The semen analysis was performed according to the 6^th^ edition of the WHO laboratory manual for examination and processing of human semen (WHO, 2021). The participants provided semen samples by masturbation after a period of abstinence from 2 to 7 days. After collection, the samples were allowed to liquefy at 37^o^C for up to 1 hour prior to analysis. Viscosity assessment was performed by allowing semen to drop by gravity and observing the length of any thread. Abnormal viscosity was determined if the drop formed a thread more than 2 cm long. Sperm counts were analyzed in a bright-field Olympus CX41 microscope (Olympus, Tokyo, Japan) at 200x and 400x final magnification. Labmate Pro pipettes were used for semen transfer throughout the procedures (HTL SA, Warsaw, Poland). Six hemocytometer chambers with improved Neubauer ruling (Qualividros, Minas Gerais, Brazil) and one Makler counting chamber (Sefi Medical Instruments, Haifa, Israel) were used for manual estimation of sperm concentrations.

### Sperm concentration measurements with the Neubauer hemocytometer

An initial microscopic examination of semen sample was performed to determine the dilution required for assessing at least 200 spermatozoa. After proper dilution, the two sides of the hemocytometer were loaded until they were full, making sure that the chamber was not underfilled or overfilled. The hemocytometer was stored horizontally for 10 - 15 minutes in a humid chamber to enable complete spermatozoa sedimentation and to prevent drying out. Sperm concentration acquired with the Neubauer hemocytometer was estimated in accordance with WHO parameters (WHO, 2021). Duplicate counts were made for each semen sample.

### Sperm concentration measurements with the Makler chamber

A well-mixed 5 µl aliquot of semen sample was placed in a water bath at 60^o^C for 5 minutes to make sure spermatozoa were immotile before counting. The heated sample was then transferred to the Makler chamber, and the glass cover was immediately applied. Care was taken to avoid bubble formation. A low magnification 100x microscopic evaluation was first performed to make sure the sample was evenly spread. Recognizable spermatozoa were counted in 10 squares with 200x magnification and results were reported as millions per ml. The entire grid (100 squares) was counted in cases of oligozoospermia (Makler, 1980). Duplicate counts were made for each semen sample.

### Latex Beads

Two standard quality-control suspensions of 4µm latex beads with target concentrations of 50±12.3 x 10^6^/ml and 25±6.5x10^6^/ml were used as reference stocks (QwikCheck Beads, Medical Electronic Systems, Los Angeles, USA). Additional stocks were prepared by diluting the 50x10^6^/ml suspension with phosphate buffered saline (PBS) to achieve final concentrations of 5x10^6^/ml, 10x10^6^/ml and 15 x10^6^/ml latex beads.

For analysis with the Neubauer hemocytometer, latex beads stock suspensions were diluted in PBS and final concentrations were estimated in accordance with the WHO manual (WHO, 2021). Well-mixed 5 µl aliquots of the 5x10^6^/ml, 10x10^6^/ml, 15x10^6^/ml, 25x10^6^/ml and 50x10^6^/ml undiluted latex beads stock suspensions were loaded and counted with the Makler chamber (MC - Und) (Makler, 1980). Each stock suspension was diluted at 1:2 with distilled water (MC - H_2_O), seminal plasma (MC - SP) and a cryopreserver (MC - Cryop) composed of egg yolk protein, glycerol, and gentamicin (FUJIFILM Irvine Scientific, Santa Ana, USA) and also counted with the chamber. All freshly prepared suspensions were manually counted with Neubauer hemocytometer and Makler chamber five times. A week later, new suspensions were prepared and counted five more times, amounting to a total of ten counts for each method.

### Statistical analysis

Difference between sperm concentration determination was checked for normal distribution using the Shapiro-Wilk test. Fixed bias was tested by one sample t-test for cases of normal distribution and a Wilcoxon signed-rank test for cases of not normal distribution. Comparison between sperm concentrations obtained with the Neubauer hemocytometer and the Makler chamber measurements was performed using limits of agreement (LOA) according to the method described by Bland & Altman (1986). Sperm concentration obtained with Neubauer hemocytometer was considered gold standard. LOA were estimated from the mean and 95% confidence interval (1.96 standard deviations) of concentration difference between the two methods (Bland & Altman, 1999). Percentage difference (100*(NH-MC)/mean) was also calculated and plotted against the mean of sperm concentration assessed by the two methods in the Bland and Altman plot. Linear regression analysis was performed to determine whether difference between measures was a function of measurement average values.

The Bartlett’s test was used to assess homogeneity of variances in latex bead counts obtained with the Neubauer hemocytometer and the Makler chamber. Welch ANOVA with Dunnett’s T3 multiple comparison post hoc test was used to determine if there were statistically significant differences between groups by testing for differences of means. Coefficients of variations (CV) and relative bias (study concentration-target concentration)/target concentration*100) were calculated as an index of precision and accuracy, respectively.

Statistical analysis was performed using the GraphPad Prism^®^ version 9.5.0 (GraphPad Software, San Diego, USA) software for Windows. A statistically significant difference was defined as *p*<0.05.

## RESULTS

### Sperm concentration measurements

From the 221 semen samples obtained in the study, nine were excluded because of azoospermia, resulting in a total of 212 semen samples examined for sperm concentration. Sperm concentration ranged from 0.07x10^6^/ml to 325.3x10^6^/ml measured with the Neubauer hemocytometer and from 0.10x10^6^/ml to 307.7x10^6^/ml measured with the Makler Chamber. The overall sperm concentration mean for each of the two counting methods is depicted in Table 1.

**Table 1:** Comparison of sperm concentration (106/ml) obtained with the Neubauer hemocytometer and Makler chamber measurements.

Sperm counting method	n	Mean (10^6^/ml)	SD
Neubauer hemocytometer	212	57.3	58.5
Makler chamber	212	58.8	58.4

n: number of samples; SD: standard deviation.

**Figure 1:**
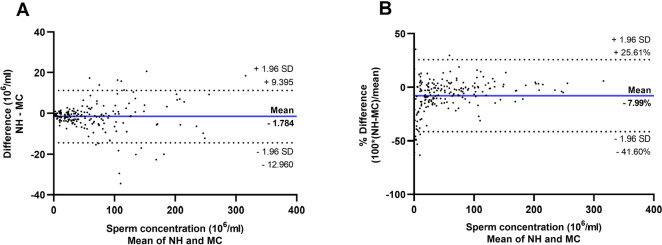
Bland and Altman plots showing mean difference (bold blue lines) and limits of agreement (mean difference ± 1.96 standard deviations (SD), dotted red lines) between sperm concentration estimated with Neubauer hemocytometer (NH) and Makler chamber (MC) as a function of (A) difference (10^6^/ml) against mean (10^6^/ml) and (B) percentage difference (%) against mean (10^6^/ml).

Bland and Altman plots are shown in Figure 1. Bias (mean difference) was estimated as -1.784x10^6^ml and limits of agreement were -12.960 and 9.395x10^6^ml for agreement analysis between sperm concentration assessment with the two methods using raw data (Figure 1A). A funnel effect can be observed in Figure 1, in which variation in difference was smaller for lower sperm concentrations and increased as values became higher. For this reason, limits of agreement were reassessed after data transformation into percentage differences (Figure 1B). Bias was estimated as -7.99% and the new limits of agreement ranged from -41.6% to 25.6%. Fixed bias was significant between analysis with the Neubauer hemocytometer and the Makler chamber (*p*<0.001).

**Figure 2:**
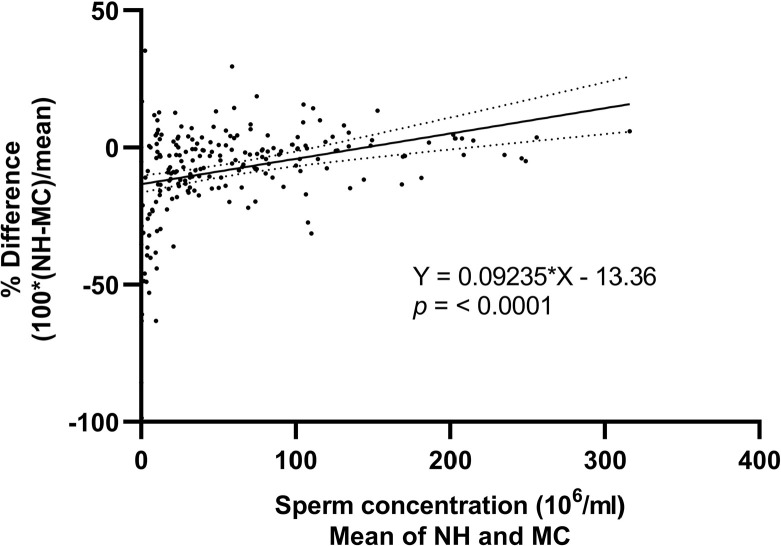
Linear regression analysis showing proportional bias between sperm estimated with Neubauer hemocytometer and Makler chamber.

It was possible to observe in Figure 1B that the Makler chamber counts yielded values that diverged progressively from those from the Neubauer hemocytometer counts across the range of results. This proportional bias was confirmed by linear regression analyses (*p*<0.001) (Figure 2). To further investigate this behavior, sperm concentrations were subdivided into < 16x10^6^/ml, 16 to 40x10^6^/ml, 40 to 80x10^6^/ml and >80x10^6^/ml based on estimations made with the Neubauer hemocytometer. Mean values for the different ranges of sperm concentration is presented in Table 2 for each counting method. A systematic difference (fixed bias) was found for concentration values inferior to 16 x 10^6^/ml (*p*<0.001) and for the 16 to 40x10^6^/ml range (*p*<0.001). Concentration results for the 40 to 80x10^6^/ml (*p*=0.052) range and for values superior to 80x10^6^/ml (*p*=0.421) were not statistically different between the two chambers.

**Table 2:** Comparison of different ranges of sperm concentration obtained with Neubauer hemocytometer and Makler chamber measurements.

Sperm concentration range (10^6^/ml)	Sperm counting method	n	Mean (10^6^/ml)	SD
< 16	NH	56	6.44	4.54
	MC	56	7.32	4.88
16 to 40	NH	54	26.9	6.78
	MC	54	28.7	6.95
40 to 80	NH	48	59.5	11.4
	MC	48	61.3	12.6
80	NH	54	138.5	55.1
	MC	54	140.1	53.8

NH: Neubauer hemocytometer; MC: Makler chamber; n: number of samples; SD: standard deviation.

Twenty-three out of 212 of samples (10.8%) presented high viscosity. To investigate whether high viscosity could influence the assessment of fixed bias, we separated normal from abnormal viscosity results. We found that 8.9% of samples with sperm concentration inferior to 16x10^6^/ml had high viscosity; while for ranges of 16 to 40x10^6^/ml, 40 to 80x10^6^/ml and > 80x10^6^/ml, high viscosity was found in 16.7%, 10.4% and in 7.4% of cases, respectively. Fixed bias was still observed when high viscosity samples were excluded from analysis for concentration values inferior to 16x10^6^/ml (*p*<0.001) and for the 16 to 40x10^6^/ml range (*p*<0.001), but not for the 40 to 80x10^6^/ml (*p*=0.077) range or concentration values superior to 80x10^6^/ml (*p*=0.639).

**Figure 3:**
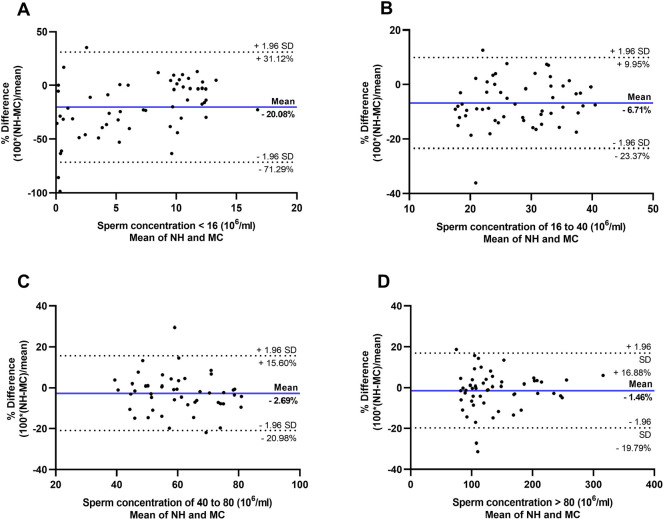
Bland and Altman plots showing mean percentage difference (bold blue lines) and limits of agreement (mean difference ± 1.96 standard deviations (SD), dotted red lines) for the (A) < 16 x 10^6^/ml, (B) 16 to 40 x 10^6^/ml, (C) 40 to 80 x 10^6^/ml and (D) > 80 x 10^6^/ml subdivisions of sperm concentration estimated with Neubauer hemocytometer (NH) and Makler chamber (MC).

Presence of fixed bias in smaller concentration ranges can be observed in the Bland and Altman plots in Figure 3. For sperm concentration values inferior to 16x10^6^/ml, the Makler chamber counts overestimated the Neubauer hemocytometer counts by a mean of -20.08%, while in the 16 to 40x10^6^/ml range, the Makler chamber counts overestimated the Neubauer hemocytometer counts by a mean of -6.71%. For higher concentrations, the percentage difference was closer to zero, with a non-significant overestimation by the Makler chamber counts of -2.69% for the 40 to 80 x 10^6^/ml range and -1.46% for values superior to 80x10^6^/ml. Outliers can be identified by points outside the standard deviation lines in the Bland and Altman plots in Figure 3. These outliers occurred in 5.34% of measurements of sperm concentrations inferior to 16 x 10^6^/ml, in 3.70% of measurements in the 16 to 40 x 10^6^/ml range, in 4.17% of measurements in the 40 to 80 x 10^6^/ml range and in 5.56% of measurements for sperm concentration values superior to 80 x 10^6^/ml.

### Internal quality assurance with latex beads measurements

The comparison of latex beads counts made with the Neubauer hemocytometer and the Makler chamber is detailed in Table 3.

**Table 3:** Comparison of different concentrations of latex beads counts obtained with Neubauer hemocytometer and the Makler chamber measurements.

Latex beads target concentration (10^6^/ml)	Counting chamber	n	Mean (106/ml)	SD	CV (%)	Relative bias (%)
5	NH	10	5.42	0.36	6.67	8.40
	MC - Und	10	6.90	1.11	16.1	38.0
	MC - H_2_O	10	5.94	1.52	25.6	18.8
	MC - SP	10	6.20	1.15	18.6	24.0
	MC - Cryop	10	6.70	0.84	12.6	34.0
10	NH	10	10.2	0.51	5.05	2.10
	MC - Und	10	12.8	1.72	13.5	27.6
	MC - H_2_O	10	11.4	0.97	8.52	14.4
	MC - SP	10	10.8	1.91	17.7	7.60
	MC - Cryop	10	11.7	1.27	10.8	16.7
15	NH	10	15.5	0.65	4.22	3.13
	MC - Und	10	18.1	2.28	12.6	21.1
	MC - H_2_O	10	16.6	1.40	8.46	10.7
	MC - SP	10	17.3	2.14	12.4	15.3
	MC - Cryop	10	17.1	1.65	9.62	14.1
25	NH	10	25.0	0.80	3.18	0.12
	MC - Und	10	27.8	1.94	6.98	11.0
	MC - H_2_O	10	29.0	3.10	10.7	15.8
	MC - SP	10	28.9	3.92	13.6	15.4
	MC - Cryop	10	27.8	3.11	11.2	11.0
50	NH	10	50.4	1.52	3.01	0.76
	MC - Und	10	54.0	3.70	6.85	8.04
	MC - H_2_O	10	53.9	5.43	10.1	7.80
	MC - SP	10	57.7	6.78	11.8	15.4
	MC - Cryop	10	55.7	5.19	9.31	11.4

NH: Neubauer hemocytometer; MC - Und: Makler chamber counts of undiluted latex beads; MC - H_2_O: Makler chamber counts of latex beads diluted with water; MC - SP: Makler chamber counts of latex beads diluted with seminal plasma; MC - Cryop: Makler chamber counts of latex beads diluted with cryopreserver; n: number of samples; SD: standard deviation; CV: coefficient of variation.

**Figure 4:**
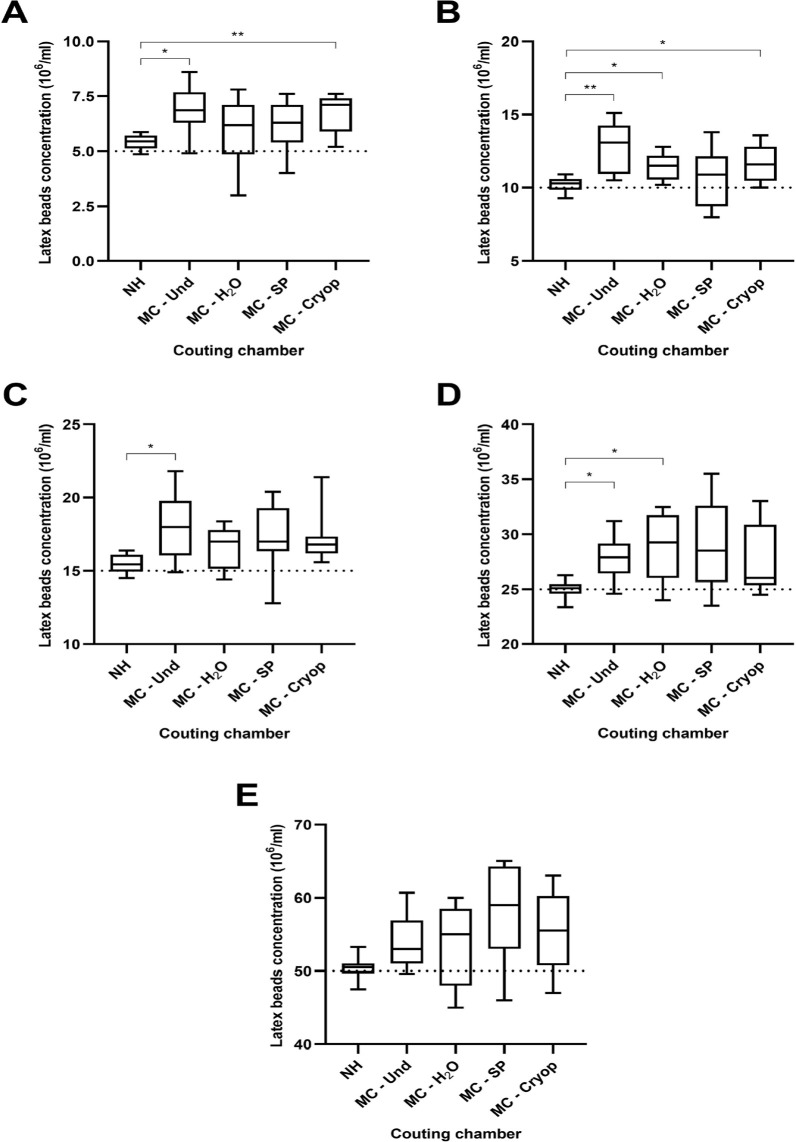
Box and whisker plot showing results of latex beads counts in Neubauer hemocytometer (NH), Makler chamber counts of undiluted latex beads (MC - Und), Makler chamber counts of latex beads diluted with water (MC - H_2_O), Makler chamber counts of latex beads diluted with seminal plasma (MC - SP) and Makler chamber counts of latex beads diluted with cryopreserver (MC - Cryop) of target concentrations of (A) 5x10^6^/ml, (B) 10x10^6^/ml, (C) 15x10^6^/ml, (D) 25x10^6^/ml and (E) 50x10^6^/ml. Box contains lower quartile (25th percentile), upper quartile (75th percentile), and median in the middle. Whiskers represent minimum and maximum. Dotted lines represent target concentration values. Significant difference between groups was determined with Welch ANOVA with Dunnett’s T3 multiple comparison post hoc test. *p*<0.05 (*); *p*<0.01 (**).

The box and whisker plots in Figure 4 reveal distribution of measurements obtained with the Neubauer hemocytometer and with the Makler chamber for each of the latex beads target values. In the 5 x 10^6^/ml target concentration (Figure 4A), there was statistical difference between analyses in the NH *vs*. MC - Und (*p*=0.0177) and between NH *vs*. MC - Cryop (*p*=0.0076) groups. In the 10x10^6^/ml target concentration (Figure 4B), there was statistical difference between the NH *vs*. MC - Und (*p*=0.0084), NH *vs*. MC - H_2_O (*p*=0.0305) and NH *vs*. MC - Cryop (*p*=0.0476) groups. In the 15 x 10^6^/ml target concentration (Figure 4C), there was statistical difference between analyses in the NH *vs*. MC - Und (*p*=0.0414) groups. In the 25 x 10^6^/ml target concentration (Figure 4D), there was statistical difference between the NH *vs*. MC - Und (*p*=0.0132) and between the NH *vs*. MC - H_2_O (*p*=0.0268) groups. No statistical difference was found between the groups in the 50x10^6^/ml target concentration (Figure 4E).

## DISCUSSION

In our study, agreement between the Neubauer hemocytometer and the Makler chamber human sperm concentration measurements was assessed to determine whether these methods are interchangeable. Furthermore, precision and accuracy of these methods were determined by measuring latex beads target concentrations for internal quality assurance. The Makler chamber measurements overestimated sperm concentrations values when compared to the Neubauer hemocytometer results, this overestimation was significantly higher for oligozoospermic samples. Accuracy was poor when latex beads undiluted stock suspensions were assessed with the Makler chamber, although diluting suspensions with seminal plasma decreased the mean difference from measurements made with the Neubauer hemocytometer. Latex beads measurements made with the Neubauer hemocytometer were more precise than those made with the Makler chamber, yielding narrower CV values between repeated measurements.

The starting point of male factor infertility investigation is semen analysis. Semen quality cannot be based solely on assessment of a single parameter, but increasing sperm concentration has been shown to influence time for pregnancy in up to 55x10^6^/ml (Slama *et al*., 2002). Hence, sperm concentration determination holds meaningful clinical value. Correct determination of sperm concentration is paramount in a clinical setting, because an inaccurate assessment may lead to misdiagnosis and misguided selection of treatment strategy (Dardmeh *et al*., 2021; Peng *et al*., 2015). However, andrology laboratories are burdened by a lack of standardization (Björndahl *et al*., 2016).

In our study, we assessed the agreement between the Neubauer hemocytometer and the Makler chamber sperm concentration measurements. The mean difference between the two methods determined with Bland and Altman plot was -1.784x10^6^ml, showing that the Makler chamber counts overestimated sperm concentration in relation to the Neubauer hemocytometer counts throughout measurements. In contrast to our findings, an investigation comparing sperm counts made with the Neubauer hemocytometer and the Makler chamber from 112 ejaculates of fertile men found that both chambers were comparable yielding similar mean concentration values (Cardona-Maya *et al*., 2008). On the other hand, overestimation obtained with the Makler chamber measurements was observed in other studies (Dardmeh *et al*., 2021; Ginsburg & Armant, 1990; Peng *et al*., 2015).

Raw data evaluation in our study demonstrated that differences increased as sperm concentration increased, featuring a funnel effect in the Bland and Altman plot. In such cases, data should be transformed, otherwise the limits of agreement will be broader apart than necessary for small concentration values and narrower than they should be for higher values (Bland & Altman, 1999). After data transformation into percentage differences, we verified that the Makler chamber counts overestimated sperm concentration results by a mean bias of -7.99% when compared to the Neubauer hemocytometer counts. Overestimation happened to a greater degree for smaller concentration values when compared to higher concentration values, which was confirmed by linear regression analyses. We then analyzed different sperm concentration ranges to determine the Makler chamber overestimation magnitude in each range. Systematic difference was found between the chambers for concentration ranges inferior to 40x10^6^/ml. Broad limits of agreement were observed for assessment in concentrations inferior to 16x10^6^/ml, especially for cases of severe oligozoospermia (sperm concentration inferior to 5x10^6^/ml). Marchlewska *et al*. (2010) found no difference between the mean concentration of spermatozoa obtained with both chambers for entire group analyses. However, after subdivision of sperm concentrations into normozoospermia (≥20x10^6^/ml) and oligozoospermia (<20x10^6^/ml), overestimation of the Makler chamber counts was observed solely in the oligozoospermia group (Marchlewska *et al.*, 2010). While sperm concentration cut-off of 20x10^6^/ml was used in this study to divide results into groups, our study inferred that the Makler chamber cannot be used interchangeably with the Neubauer hemocytometer when initial concentration analyses render results inferior to 40 x 10^6^/ml. Since lower sperm concentration results are the most valuable for clinical decisions, reliable determinations are indispensable.

Many laboratories that include semen analyses in their routine are burdened by a lack of standardization (Björndahl *et al*., 2016). It is essential that internal quality assurance practices are installed to guarantee precise and accurate determinations. Imade *et al*. (1993) reported that those assessments made with the Makler chamber showed better precision results when compared to the Neubauer hemocytometer measurements, with a CV of 5.9% and 7.1%, respectively. Moreover, the authors recommended using the Makler chamber because it gave midway sperm concentration values, while the Neubauer hemocytometer underestimated results (Imade *et al*., 1993). However, there is no guarantee that midway values found with the Makler chamber measurements are correct. An approach to amend this problem is by measuring either known spermatozoa concentrations or commercially available standard latex beads.

In our study, we employed commercially available standard latex beads for internal quality assurance. We observed that the Neubauer hemocytometer counts were more precise than the Makler chamber counts, with a CV varying from 3.01% to 6.67%, while for the Makler chamber, the CV varied from 8.46% to 25.64%. Similar to our results, an external quality assurance program in Australia found a CV of 14.6% for the Neubauer hemocytometer and a CV of 24.1% for the Makler chamber measurements (Zuvela & Matson, 2020). When assessing repeated counts of sperm samples, the Neubauer hemocytometer demonstrated the lowest CV (8.50%) and the Makler chamber the highest (13.1%) (Bailey *et al*., 2007).

We also found that measurements of undiluted stock suspensions with the Makler chamber overestimated latex beads concentrations to a greater degree than the Neubauer hemocytometer for target concentrations up to 25x10^6^/ml. Similarly, to our observation regarding higher sperm concentrations in semen samples, there was no difference between chambers when the 50 x 10^6^/ml latex beads stock suspension was analyzed. Ginsburg & Armant (1990), verified that bead counts obtained with the Neubauer hemocytometer were not statistically different from those determined by electronic particle counting; whereas the Makler chamber counts were 62% higher (Ginsburg & Armant, 1990). We did not find a difference of this magnitude from target concentrations with the Makler chamber in our investigation. The biggest difference we found was of 38% by assessing the undiluted stock suspension of 5x10^6^/ml.

Variation between results may be attributed to differences in chamber depth and sampling error. While the Neubauer hemocytometer has a depth of 100 µm, the Makler chamber has a depth of 10 µm. Thus, small changes because of improper placing of the cover glass could result in greater proportional differences in the shallower chamber. Moreover, when 200 beads are counted in the entire Makler chamber, the resulting concentration will be 20x10^6^/ml. Hence, for concentration values below this threshold, fewer than 200 beads will be counted, which increases the sampling error (Peters *et al*., 1993; WHO, 2021). We observed this phenomenon for lower latex beads concentrations for values up to 10x10^6^/ml showed the highest CV and relative bias in our study.

Delaying application of the Makler chamber cover glass can also be a potential source of error with sperm concentrations progressively increasing with a longer delay. The medium used to suspend beads should also be taken into consideration. Matson *et al*. (1999) observed that non-physiologic PBS was the worst choice for Makler chamber assessment because beads settled very quickly resulting in overestimation. However, diluting beads with serum or seminal plasma allowed them to settle the least over the first 10 seconds (Matson *et al*., 1999). Mahmoud *et al*. (1997) similarly found that mixing bead suspensions with seminal plasma led to correct estimation of bead concentration. In our study, dilution with PBS showed the worst precision indexes for analysis made with the Makler chamber. The Makler chamber mean values were also significantly different from measurements made with the Neubauer hemocytometer for suspensions up to 25x10^6^/ml diluted with PBS.

The Makler chamber assessment of suspensions diluted with water and cryopreserver revealed statistically different means from the Neubauer hemocytometer results for target concentrations of 5x10^6^/ml, 10x10^6^/ml and 25x10^6^/ml. In accordance with other studies (Mahmoud *et al*., 1997; Matson *et al*., 1999), beads suspensions diluted with seminal plasma assessed with the Makler chamber was the only not showing statistical difference from measurements made with the Neubauer hemocytometer. Although the CV were still unacceptably high for all target concentrations.

A few outliers (differences outside limits of agreements) were observed in the limits of agreement study between the Neubauer hemocytometer and the Makler chamber. Occasional outliers are to be expected considering that inherent semen viscosity and sampling errors can contribute to analytical variability between measurements. Bailey *et al*. (2007) found that 3.68% of measurements were outliers when testing for agreement between the two chambers, while we observed 10 outliers out of 212 samples (4.72%). These extreme readings departing from the mean difference values in our study occurred evenly throughout different sperm concentration ranges, possibly caused by analytical errors. Furthermore, we used negative displacement pipettes in our study because positive displacement pipettes recommended by the WHO manual (WHO, 2021) were not available in our laboratory. We found that samples with high viscosity did not influence our results, and eliminating those samples did not eradicate systematic difference between measurements made with the Neubauer hemocytometer and the Makler chamber for sperm concentrations inferior to 40x10^6^/ml. Thus, although diluting latex beads with seminal plasma during quality assurance evaluation was able to decrease the difference between chamber results, analysis of real semen samples was not. We believe this was due to the heterogenous nature of different semen samples analyzed in the method agreement study, while a single sample was used to produce seminal plasma for dilution of latex beads in the quality assurance study.

## CONCLUSIONS

Agreement between the Neubauer hemocytometer and the Makler chamber measurements was poor for sperm concentration values inferior to 40x10^6^/ml. Our results also revealed that concentration measurement with Neubauer hemocytometer was confirmed as the most precise and accurate method for concentration assessment, while measurements made with the Makler chamber demonstrated more variability and a higher degree of overestimation. Sperm concentration assessment made with the Makler chamber comes with the risk of overestimating results, especially for oligozoospermic samples. Andrology laboratories should clearly state which method is being employed for sperm concentration assessment, so clinicians can be made aware of measurement uncertainty when using the Makler chamber for infertility investigation in men.

## Data Availability

The data would be available upon request.
